# SU(VAR)3-7 Links Heterochromatin and Dosage Compensation in Drosophila

**DOI:** 10.1371/journal.pgen.1000066

**Published:** 2008-05-02

**Authors:** Anne Spierer, Flora Begeot, Pierre Spierer, Marion Delattre

**Affiliations:** NCCR “Frontiers in Genetics”, Department of Zoology and Animal Biology, University of Geneva, Geneva, Switzerland; European Molecular Biology Laboratory, Germany

## Abstract

In Drosophila, dosage compensation augments X chromosome-linked transcription in males relative to females. This process is achieved by the Dosage Compensation Complex (DCC), which associates specifically with the male X chromosome. We previously found that the morphology of this chromosome is sensitive to the amounts of the heterochromatin-associated protein SU(VAR)3-7. In this study, we examine the impact of change in levels of SU(VAR)3-7 on dosage compensation. We first demonstrate that the DCC makes the X chromosome a preferential target for heterochromatic markers. In addition, reduced or increased amounts of SU(VAR)3-7 result in redistribution of the DCC proteins MSL1 and MSL2, and of Histone 4 acetylation of lysine 16, indicating that a wild-type dose of SU(VAR)3-7 is required for X-restricted DCC targeting. SU(VAR)3-7 is also involved in the dosage compensated expression of the X-linked *white* gene. Finally, we show that absence of maternally provided SU(VAR)3-7 renders dosage compensation toxic in males, and that global amounts of heterochromatin affect viability of ectopic MSL2-expressing females. Taken together, these results bring to light a link between heterochromatin and dosage compensation.

## Author Summary

In Drosophila, females have two X chromosomes and males only one. The difference in the dose of X-associated genes is compensated by male-specific protein machinery, the Dosage Compensation Complex (DCC), which augments the activity of genes of the single male X. We report that the specific targeting of the DCC on the male X chromosome depends critically on the correct dose of the SU(VAR)3-7 protein. This protein was previously known to associate with condensed and silenced regions of the chromosomes called heterochromatin by contrast with the active form of chromatin called euchromatin. Loss of SU(VAR)3-7 in males causes displacement of the DCC to heterochromatin and bloating of the X chromosome. In contrast, excess of SU(VAR)3-7 leads to a delocalization of the DCC to other chromosomes and to massive shrinking of the X chromosome. We show that SU(VAR)3-7 is involved in the dosage compensated expression of the X-linked *white* gene and in the viability of dosage compensated flies. Altogether, these results bring to light a link between silencing mechanisms of heterochromatin and mechanisms controlling the balance of sex-chromosome activity (dosage compensation). This opens new perspectives on how complexes that control the global chromosome organisation impact the fine tuning of gene expression.

## Introduction

*Drosophila melanogaster* uses two systems of whole chromosome regulation: dosage compensation mediating the two fold up-regulation of male X-linked genes and the Painting of Fourth, POF, regulating the mainly heterochromatic fourth chromosome. Binding of POF to the fourth chromosome is dependent on the heterochromatic protein HP1 [1]. POF and HP1 colocalize on fourth chromosome-linked genes and both are involved in the global regulation of the fourth chromosome [1],[2]. Johansson et al. (2007) proposed that POF stimulates and HP1 represses genes expression and that the interdependent binding of these two proteins precisely tunes output from the fourth chromosome.

Dosage compensation targets the male X chromosome to correct the unbalance between the unique X chromosome of males and the two X chromosomes of females. To compensate for the resulting disparity in X chromosome-linked gene expression, most X-linked genes in males are hyperactivated. The Dosage Compensation Complex (DCC) consists of five proteins called the MSLs for Male Specific Lethal (MSL1, MSL2, MSL3, MLE and MOF) as well as two non-coding RNAs, roX1 and roX2 (reviewed in [3],[4],[5],[6]). In males, the expression of MSL2 mediates the stabilization of the other proteins and the assembly of the DCC specifically on the X chromosome [7]. This results in an enrichment of acetylation of histone H4 at lysine 16 (H4K16ac) on the male X chromosome, due to the MOF protein of the complex [8],[9]. The histone mark could in part explain the subsequent hypertranscription of X-linked genes in males [10],[11]. In females, the *Sex-lethal* gene turns off the dosage compensation system by repressing the *Msl2* translation [12],[13].

One of the most intriguing issues of dosage compensation is the specific recognition of the male X chromosome by the DCC. Searches for X chromosomal DNA sequences determining DCC binding failed to identify a consensus sequence [14],[15],[16]. Global mapping of MSL proteins on the X chromosome has demonstrated that the DCC associates primarily with genes rather than intergenic regions, displays a 3′- bias and correlates with transcription [14],[16],[17]. Moreover, the MSL complex is attracted to genes marked by H3K36 trimethylation, a mark of active transcription [18]. Furthermore, the levels of DCC proteins MSL1 and MSL2 are critical for correct targeting to the X chromosome [19]. Over-expression of both *msl1* and *msl2* results in inappropriate MSLs binding to the chromocenter and chromosome 4 [19],[20]. MSL2, deleted of its C-terminal part, binds as a complex with MSL1 to the heterochromatic chromocenter [21]. *roX* RNAs are also key components for X chromosome targeting since *roX1roX2* mutants cause relocation of MSLs complex to autosomal regions and the chromocenter [22],[23]. These data reveal an unpredicted physical association between the MSL complex and heterochromatic regions.

H4K16 acetylation is not the only chromatin mark distinguishing the Drosophila male X chromosome from the autosomes. Phosphorylation of H3 at serine 10, catalyzed by JIL-1, is a histone modification highly enriched on the male X chromosome [24]. The JIL-1 kinase interacts with the DCC and is involved in dosage compensation of X-linked genes [25],[26]. Interestingly, *Jil-1* mutant alleles affect both condensation of the male X chromosome and expansion of heterochromatic domains, providing evidence for a dynamic balance between heterochromatin and euchromatin [27],[28]. Other general modulators of chromatin state, as ISWI or NURF, are also required for normal X chromosome morphology in males [29],[30],[31]. The NURF complex and MSL proteins have opposite effects on X chromosome morphology and on *roX* transcription [32].

We have discovered previously an intriguing genetic interaction between the heterochromatic proteins SU(VAR)3-7 and HP1, and dosage compensation [33]. *Su(var)3-7* encodes a protein mainly associated with pericentromeric heterochromatin and telomeres, but also with a few euchromatic sites [34],[35],[36]. Specific binding to pericentric heterochromatin requires the heterochromatic protein HP1 [33]. HP1 localizes to heterochromatin through an interaction with methylated K9 of histone H3 (H3K9me2), a heterochromatic mark mainly generated by the histone methyltransferase SU(VAR)3-9 [37],[38],[39]. SU(VAR)3-7 interacts genetically and physically with HP1 [35],[36] and with SU(VAR)3-9 [38],[40]. *Su(var)3-7*, *Su(var)2-5* encoding HP1 and *Su(var)3-9* are modifiers of position effect variegation (PEV), the phenomenon of gene silencing induced by heterochromatin [34],[41],[42] (reviewed in [43],[44]). These three genes enhance or suppress the PEV depending on their doses and thus are considered as encoding structural components of heterochromatin [45]. Strikingly, the amounts of SU(VAR)3-7 and HP1 affect male X chromosome morphology more dramatically than other chromosomes. Reduced doses of SU(VAR)3-7 or HP1 result in bloating of the X chromosome specifically in males [33]. Increased doses of SU(VAR)3-7 cause the opposite phenotype, a spectacular condensation of the X chromosome associated with recruitment of other heterochromatin markers [40]. Some unique feature of the male X chromosome makes it particularly sensitive to change in SU(VAR)3-7 amounts. In addition, knock-down of *Su(var)3-7* results in reduced male viability leading to a 0.7 male/female ratio in the progeny of *Su(var)3-7* homozygous mutant mothers [40]. The possibility of interaction between activating and repressive chromatin factors on the male X chromosome led us to analyze the impact of SU(VAR)3-7 on dosage compensation.

In this study we show that wild-type levels of SU(VAR)3-7 are required for male X chromosome morphology, X chromosome-restricted DCC targeting, expression of P(*white*) transgenes in males and for coping with increased MSL1 and MSL2 levels. We provide evidence for interplay between heterochromatin and dosage compensation in Drosophila.

## Results

### The DCC Makes the X Chromosome a Preferential Target for Heterochromatinization by *Su(var)3-7* Over-Expression

**Figure 1:**
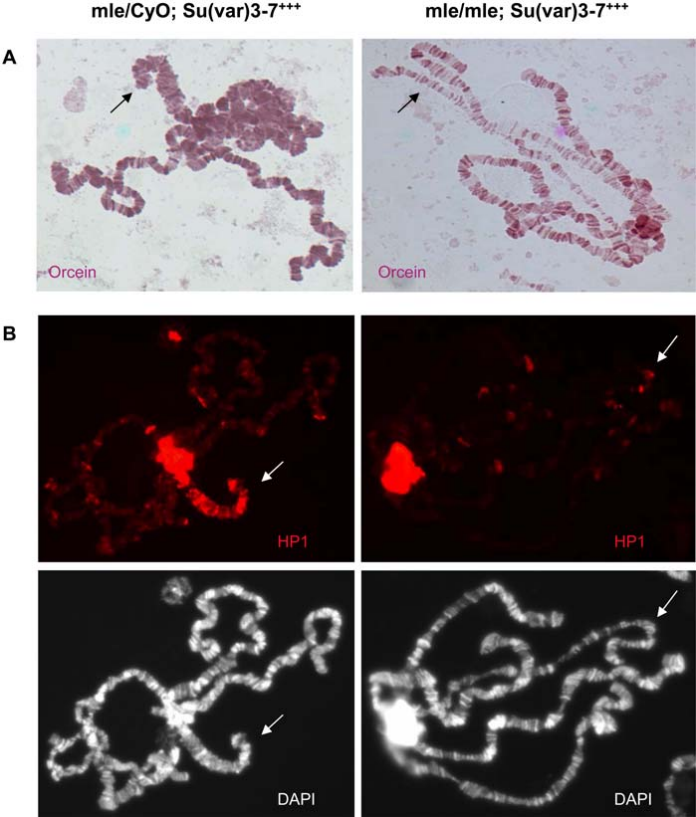
Severe condensation of the X chromosome by increased *Su(var)3-7* expression requires the Dosage Compensation Complex. Polytene chromosomes from male third instar larvae containing a heat-shock transgene over-expressing *Su(var)3-7* (Su(var)3-7^+++^) in combination with the heterozygous or homozygous *mle^1^* mutation. A: Orcein staining. B: Immunodetection of HP1 (red) and DNA staining with DAPI. Larvae were submitted to daily heat-shocks at 35°C before squashing. Arrows indicate the X chromosome.

An excess of SU(VAR)3-7 induces male and female lethality and causes spectacular changes in the morphology of polytene chromosomes [40]. The male X chromosome is always the most affected chromosome: it becomes highly condensed and shortened and its characteristic banding pattern is modified. To test whether the Dosage Compensation Complex is required for the male X chromosome phenotypes, we over-expressed *Su(var)3-7* in a mutant for the DCC and we examined the morphology of the X chromosome (Figure 1). Homozygous mutants in *mle*, the gene encoding the RNA helicase component of the DCC, do not compensate for dose and die at the third-instar larval stage, late enough to permit examination of polytene chromosomes [46],[47]. The combination of *mle^1^* with a transgene over-expressing *Su(var)3-7* (P[HA: SuvarFL4D] [48]), results in an almost normal male X chromosome morphology whereas brothers in the same progeny heterozygous for the *mle^1^* mutation still display strong condensation of the X chromosome (Figure 1A). Thus, the condensation process of the male X chromosome in presence of an excess of SU(VAR)3-7 requires the Dosage Compensation Complex. We had shown previously that the male X chromosome condensation coincided with association of SU(VAR)3-7 all along the X chromosome and recruitment of the heterochromatic proteins HP1 and H3K9me2 [40]. To test whether the enrichment of heterochromatic markers on the male X chromosome requires the presence of the DCC, we performed immunostaining on male larvae expressing the *Su(var)3-7* transgene together with the *mle^1^* mutation. In homozygous *mle^1^* larvae over-expressing *Su(var)3-7*, SU(VAR)3-7, HP1 and H3K9me2 enrichment on the male X chromosome is lost in contrast to the heterozygous *mle* brothers of the same cross (not shown, and Figure 1B). We conclude that association of the DCC on the X chromosome is required to make the X chromosome a preferential target for heterochromatic markers in a context of high levels of SU(VAR)3-7.

**Figure 2:**
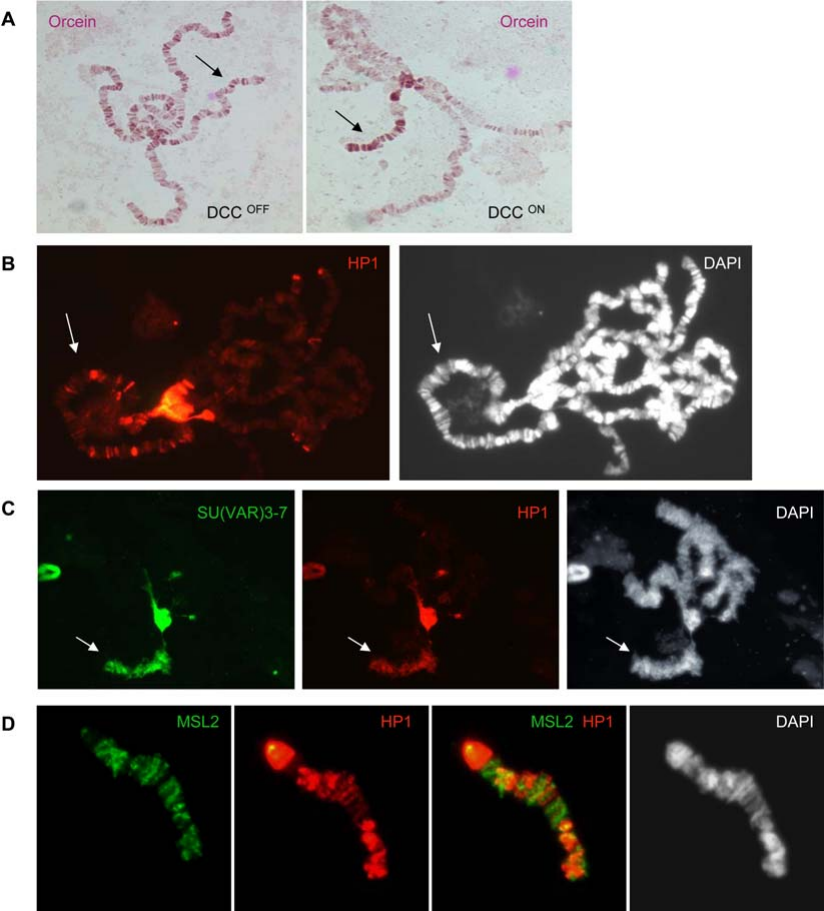
Presence of the Dosage Compensation Complex is sufficient for the X chromosome condensation resulting from *Su(var)3-7* over-expression. Polytene chromosomes from female third instar larvae harbouring the heat-shock transgene over-expressing *Su(var)3-7* with (DCC^ON^) or without (DCC^OFF^) the transgene expressing MSL2. A: Orcein staining. B and C: Immunodetection of HP1 (red) and SU(VAR)3-7 (green) on dosage-compensated females over-expressing *Su(var)3-7* (B: moderate *Su(var)3-7* expression (one daily heat-shock) and C: strong *Su(var)3-7* expression (three daily heat-shocks at 35°C)). Arrows indicate the X chromosomes. D: Double immunodetection of MSL2 (green) and HP1 (red) on X chromosomes in SU(VAR)3-7 excess condition.

Then, to test whether the DCC is not only necessary but also sufficient for the altered morphology of the X chromosome, we examined X polytene chromosomes of females expressing the *Su(var)3-7* transgene and the DCC (Figure 2). Dosage compensation in females was artificially induced by a transgene expressing MSL2 under the control of the hsp83 promoter [7]. The expression of *msl-2* in females over-expressing *Su(var)3-7* causes the X chromosomes to condense as typically seen only in males over-expressing *Su(var)3-7* (Figure 2A). Furthermore, assembly of the DCC on the X chromosomes in these females leads to an enrichment of SU(VAR)3-7 binding on the X chromosomes, but also of HP1 and H3K9me2: the heterochromatic markers enrichment on the X chromosomes is more or less abundant according to the strength of the heat-shock inducing expression of *Su(var)3-7* (Figure 2 B, 2C, and not shown). This X chromosomes coating by heterochromatic markers is never observed in wild type females (not shown and [40]). The Dosage Compensation Complex is therefore not only necessary but also sufficient to allow the massive recruitment of heterochromatic proteins on the X chromosomes induced by high levels of SU(VAR)3-7.

On the condensed male or female X chromosomes, MSL2 does not colocalize with the heterochromatic proteins: some regions of the X chromosome enriched for SU(VAR)3-7 and HP1 are almost devoid of MSLs and inversely (Figure 2D). The dosage compensation complex renders the X chromosomes especially attractive to the SU(VAR)3-7/HP1 complex, when in large amounts, but its binding pattern differs from that of MSLs.

### A Lack of Maternal SU(VAR)3-7 Protein Reduces *White* Expression Specifically in Males

**Figure 3:**
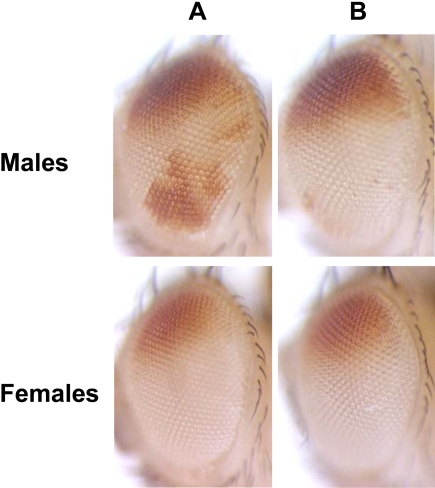
Lack of SU(VAR)3-7 maternal product decreases mini-*white* expression in males. Mini-*white* expression at 25°C in eyes of P[w^+^GMroX1] adult flies harbouring the transgene at cytological location 69C [50]. Males and females are hemizygous for the P[w^+^GMroX1]69C insertion and are issued either from (A) wild type *w^1118^* mothers or (B) homozygous *Su(var)3-7^R2a8^* females. The lack of maternal SU(VAR)3-7 product represses *white* expression in the ventral part of the eyes in males but not in females. Flies were observed five days after hatching.

To test whether SU(VAR)3-7 is recruited by the DCC, we examined whether a P[w^+^GMroX1] transgene, known to recruit the DCC when inserted on an autosome [49], is able to attract SU(VAR)3-7 and HP1 at its insertion site. Three P[w^+^GMroX1] insertions (at 85D, 69C, 79B [50]) were tested by immunostaining on males salivary gland polytene chromosomes, with antibodies against MSL2 as a control for efficient DCC assembling at the insertion site and against SU(VAR)3-7 and HP1 for the creation of a new binding site at these locations. Although strong MSL2 staining was detected at the autosomal site of the three lines, neither SU(VAR)3-7 nor HP1 were detected at these cytological locations (not shown). We conclude that the DCC binding to the *roX1* transgene does not recruit detectable amounts of SU(VAR)3-7 and HP1 proteins. In addition, we crossed the transgenic P[w^+^GMroX1] males with wild type females, or females homozygous mutants for *Su(var)3-7*, in order to test whether reduced SU(VAR)3-7 amounts modify the extent of MSLs spreading around the insertion [49]. We did not observe significant changes of the local MSL spreading in the absence of maternal SU(VAR)3-7 (not shown). However, we were surprised to note that the expression of the *white* gene, a reporter gene within the transgene, was modified by the absence of maternal SU(VAR)3-7 product in the three lines (Figure 3 and not shown). The well-known X-linked *white* gene and its truncated version mini*-white* retain full dosage compensation on the X and partial dosage compensation when transposed to autosomes [51]. Our observation that *white* expression is specifically reduced in males in the crosses that lack maternal SU(VAR)3-7 leads us to conclude that SU(VAR)3-7 specifically regulates *white* expression in males. This suggests an implication of SU(VAR)3-7 in dosage compensation.

### Wild-Type Levels of SU(VAR)3-7 Are Required To Restrict DCC Binding to the X Chromosome

**Figure 4:**
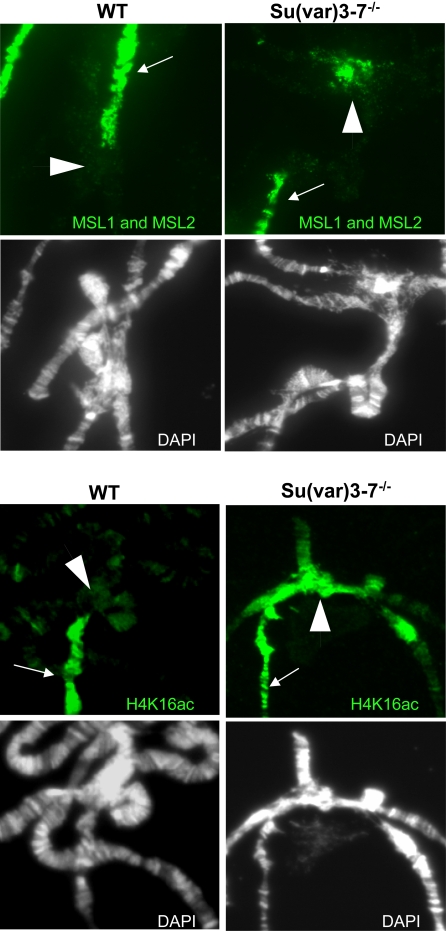
The Dosage Compensation Complex is delocalized to the chromocenter of *Su(var)3-7* mutant polytene chromosomes. Immunodetection of MSL1 and MSL2 or H4K16ac on wild-type males (WT) or *Su(var)3-7^9/9^* males raised at 29°C (*Su(var)3-7^−/−^*). Arrows indicate the X chromosome and arrowheads show the chromocenter. Complete chromosomes spreads are shown in Figure S1.

We wondered then whether the wild type dose of SU(VAR)3-7 is required for correct MSLs localization. We have shown that in hypomorphic *Su(var)3-7* mutants, MSLs staining appears globally unmodified on the bloated X chromosome ([33] and Figure 4); MSL1, MSL2 and H4K16ac are still present at hundreds of sites on the male X chromosome and at very few sites on autosomes. Interestingly, in more severe *Su(var)3-7* mutant conditions, we did detect changes in MSLs localization: in *Su(var)3-7^R2a8^* or in *Su(var)3-7^9^* homozygous mutant larvae raised at 29°C, MSL1 and MSL2 proteins are clearly visible at the chromocenter in proportions that are never observed in wild-type males raised in the same condition (Figure 4 and Figure S1). The enrichment at pericentromeric heterochromatin is also visible for H4K16ac in *Su(var)3-7* mutant larvae, meaning that the MSL complex delocalized at heterochromatin is enzymatically active (Figures 4 and Figure S1). These results show that reducing the amounts of SU(VAR)3-7 delocalizes the MSLs towards heterochromatin.

**Figure 5:**
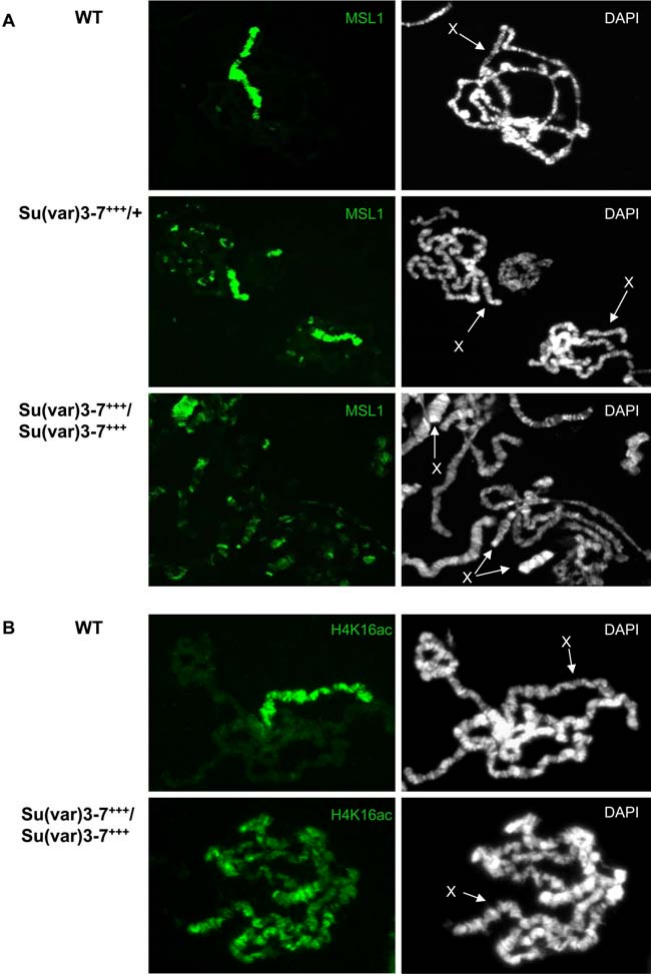
The Dosage Compensation Complex is delocalized to autosomal sites on polytene chromosomes over-expressing *Su(var)3-7*. A: MSL1 immunodetection on chromosomes harbouring zero (WT), one (Su(var)3-7^+++^/+) or two copies (Su(var)3-7^+++^/Su(var)3-7^+++^) of the heat-shock transgene expressing *Su(var)3-7*. Male larvae were submitted to daily heat-shocks at 35°C before squashing. Arrows indicate the X chromosomes. B: H4K16ac immunodetection on chromosomes of wild type males or males harbouring two copies of the heat-shock transgene over-expressing *Su(var)3-7* (Su(var)3-7^+++^/Su(var)3-7^+++^). Male larvae were submitted to daily heat-shocks at 35°C before squashing.

Next, we compared MSLs staining in presence of increased levels of SU(VAR)3-7. In wild-type males, the MSL1 protein accumulates at hundreds of sites on the X chromosome and is associated with 5 +/− 1 sites on autosomes (Figure 5). The number of autosomal sites increases to thirty on polytene chromosomes from heat-shocked larvae containing one copy of the *Su(var)3-7* transgene. With two copies of the transgene, the number of autosomal sites reaches a hundred, and MSL1 exhibits a decreased affinity for the X chromosome (Figure 5). Similar delocalization on autosomes is visible for MSL2 and H4K16ac (not shown and Figure 5). This indicates that the complex is still enzymatically active on autosomes. Staining by MSL1, MSL2 or H4K16ac of the chromocenter and of chromosome 4 is not detected. For the three proteins, the delocalization on autosomal sites is proportional to the dose of SU(VAR)3-7. As controls, absence of heat-shock in the *Su(var)3-7* homozygous transgenic line and heat shock in wild-type did not cause MSLs nor H4K16ac delocalization (Figure 5 and not shown). In sum, high levels of SU(VAR)3-7 in males lead to recruitment of heterochromatic proteins on the X chromosome and concomitantly to delocalization of the MSLs on autosomes, suggesting an antagonism between heterochromatin and the DCC. We conclude that wild-type levels of SU(VAR)3-7 are required for X chromosome**-**restricted binding of the MSLs.

### Mutation of *Su(var)3-7* Does Not Regulate the Expression of Components of the DCC

**Figure 6:**
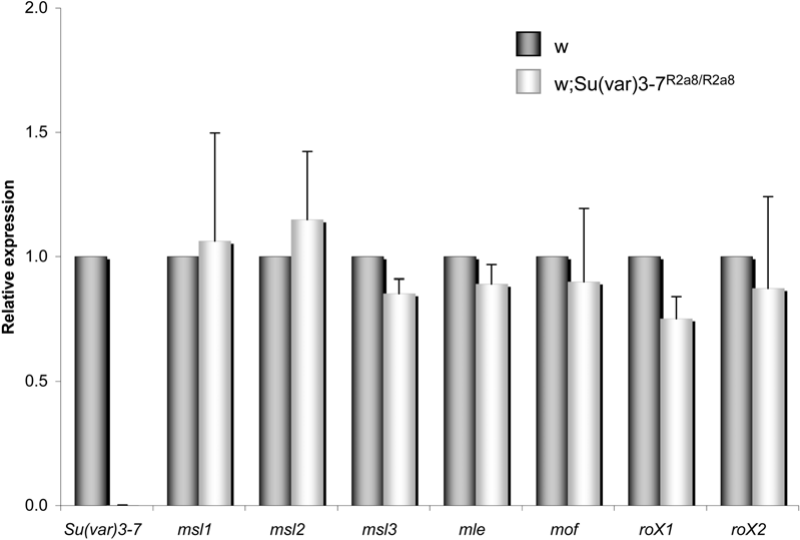
*Su(var)3-7* mutation does not modify transcript accumulation of DCC components. Histograms display quantitative RT-PCR analysis of transcript amounts in *Su(var)3-7^R2a8^* and wild-type male third instar larvae. Triplicate PCRs were performed on triplicates of samples, and the results obtained for each tested genes were normalized with four control genes (Materials and Methods). Bars represent standard deviation from the mean.

Phenotypes of MSLs relocation on autosomes or at chromocenter due to changes in SU(VAR)3-7 levels resemble those due to changes in levels of the MSLs [19],[20]. *Su(var)3-7* mutations could therefore lead to the production of an altered DCC by modifying expression of genes encoding components of the complex. We first compared MSL1 and H4K16ac levels in wild-type and *Su(var)3-7* mutant third instar larvae by Western blot analysis. We did not detect changes (not shown). We have also tested by quantitative reverse-PCR the level of transcription of *msl1, msl2, msl3, mof, mle, roX1* and *roX2* genes in wild-type and *Su(var)3-7* male mutant larvae. We did not detect either significant changes in the levels of transcription of any of these genes (Figure 6). This indicates that SU(VAR)3-7 does not regulate directly the expression of dosage compensation genes, but rather acts at the level of the X chromosome chromatin conformation.

### *Su(var)3-7* Over-Expression Affects *Mini-White* Gene Expression Specifically in Males and on the X Chromosome

We then addressed the question of whether the striking displacement of the DCC on autosomes in presence of an excess of SU(VAR)3-7 modifies the level of expression of the dosage-compensated *white* gene when located either on the X chromosome or on autosomes. The *white* gene harboured by P transgenes can indeed easily be moved to different places in the genome while conserving its dosage compensated expression [51]. *white* expression was monitored by the levels of eye pigments. Females homozygous for a P(mini*-white*) transgene (described in Materials and Methods) were crossed either to wild type males or to males harbouring the heat-shock transgene over-expressing *Su(var)3-7*. F1 progeny from both crosses was submitted to daily heat-shocks at 35°C from third instar larval stage to adulthood. We tested 12 lines harbouring the P(mini*-white*) transgene: six out of twelve contain a transgene on the X chromosome, and in the six others the transgene is on an autosome.

**Figure 7:**
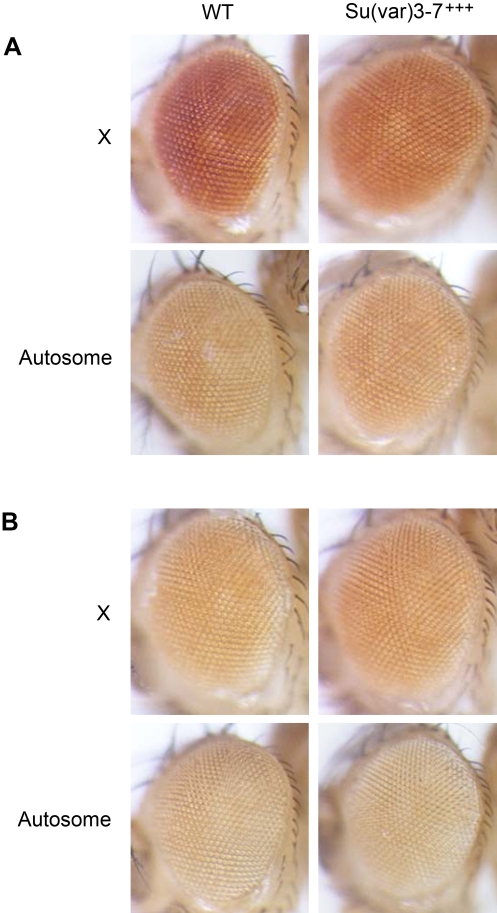
Expression of X-linked mini-*white* transgenes is specifically reduced in males by increased *Su(var)3-7* expression. P(mini*-white*) transgene expression inserted on the X or on autosomal chromosomes was studied in males (A) and females (B) in absence (WT) or presence of the heat-shock transgene overexpressing *Su(var)3-7* (Su(var)3-7^+++^). The figure displays the eyes of adult flies, submitted to daily heat-shock during their development, from two lines: one with the P(mini-*white*) transgene on the X and the other on an autosome. All the flies were submitted to the same heat-shock conditions. Flies were observed five days after hatching.

Interestingly, for none of the six lines containing the P(mini*-white*) on autosomes was eye colour modified in male or female by increased *Su(var)3-7* expression compared to wild type dose of SU(VAR)3-7 (an example is given in Figure 7). In contrast, for five out of six lines harbouring the P(mini*-white*) on the X chromosome, F1 males displayed lighter eyes in presence of over-expressed *Su(var)3-7* than in wild-type context. On the other hand, female eye colour from these crosses did not change with the dose of SU(VAR)3-7 (Figure 7). As control, we verified that repression of *white* gene never occurs in not heat-shocked crosses nor in crosses with wild-type dose of SU(VAR)3-7 submitted to daily heat-shocks (not shown and Figure 7). As for the sixth P(mini*-white*) line on the X, overproduction of SU(VAR)3-7 in this line killed the males, whereas females were perfectly viable. This unexplained male lethality prevented us to conclude on the effect of over-expressing *Su(var)3-7* on male eye colour. In sum, these data show that an increased dose of SU(VAR)3-7 reduces specifically in males the level of expression of the dosage compensated *white* gene, when and only when this gene is on the X chromosome.

The remarkable effect of SU(VAR)3-7 on the dosage compensated *white* gene led us to wonder whether SU(VAR)3-7 is required for dosage compensation of other *X*-linked genes. Expression of seven X-linked genes and two autosomal genes were analyzed by quantitative RT-PCR in third instar larvae of wild type and *Su(var)3-7* null mutant male. We analyzed transcripts levels of the seven dosage-compensated genes *arm, BR-C, CG14804, dspt6, Gs2, Pgd, mRpL16* and of two autosomal genes, *RNApolII* and *tubulin α*. The levels of transcripts were normalized to *efg1* and *gapdh* autosomal genes as internal standard. We did not detect significant modifications of expression of these X-linked genes in *Su(var)3-7* mutant males larvae compared to autosomal genes (Figure S2). These results indicate that the lack of SU(VAR)3-7 does not significantly modify the transcription level of a set of X-linked genes.

### SU(VAR)3-7 Is Required for Male Viability when MSLs Are Over-Produced

To investigate further the implication of SU(VAR)3-7 on dosage compensation, we wondered whether the amounts of SU(VAR)3-7 have an impact on male viability. Indeed, knock-down of *Su(var)3-7* results in significantly more lethality in males than in females [52]. It is however difficult to compare male and female viability as they differ by many factors as sex differentiation, dosage compensation, heterochromatin amounts, etc. We therefore examined viability among males with modified DCC activities. In a *Su(var)3-7* mutant background, we compared the viability of males in presence of different amounts of MSLs proteins by using transgenes expressing MSL1 and MSL2 under the control of the hsp83 promoter [7],[53]: The transgenes do not affect the viability of otherwise wild-type males (Table 1). But the absence of maternal SU(VAR)3-7 kills the males harbouring the transgenes: homozygous *Su(var)3-7^R2a8^* females crossed with transgenic (*H83MSL1-H83MSL2*) males produce only 6% of male adult progeny harbouring the transgenes compared to 48% if the mothers are heterozygous for a *Su(var)3-7* mutation. However, with mothers homozygous mutant for another heterochromatic component, the SU(VAR)3-9 histone-methyl-transferase, we found no effect on the viability of males. The absence of maternal SU(VAR)3-7 product specifically renders increased MSL1 and MSL2 expression toxic to males.

**Table 1:** SU(VAR)3-7 requirement for male viability in excess of MSL1 and MSL2.

Father:	F1			
H83MSL1-H83MSL2/+	with (+) or without (−) H83MSL1-H83MSL2			
X Mother:	% Male (−)	% Male (+)	% Female (−)	% Female (+)
wt	51% (525)	49% (499)	100% (561)	0% (0)
*Su(var)3-7 ^R2A8/+^*	52% (417)	48% (384)	100% (444)	0% (0)
*Su(var)3-7 ^R2A8/R2A8^*	**94%** (336)	**6%** (21)	100% (564)	0% (0)
*Su(var)3-9 ^17/17^*	51% (279)	49% (270)	100% (339)	0% (0)

### Global Amounts of Heterochromatin Affect Viability of Females Engineered To Expressing *msl2*

In Drosophila, the Y chromosome is 20 Mbases long and is made almost entirely of heterochromatin [54]. To test whether the global amount of heterochromatin has an influence on the DCC activity, we crossed either wild-type females or females engineered to expressing *msl2* by carrying the *msl2* transgene (*H83MSL2* [7]) to males bearing a compound X-Y chromosome (*C(1:Y)yw*). These crosses produce males lacking the Y chromosome (X/0) and females containing a Y chromosome (XXY). Details of the crosses and of their offspring are given in Materials and Methods and in Supporting Information Table S1. In the control crosses, female viability is slightly modified by the presence of a Y chromosome (female/male ratio: 0.72) and viability of XX females is not altered by the presence of the *msl2* expressing transgene thanks to a mutation into the endogenous *msl1* gene (female/male ratio 0.96). However the combination of the *msl2* transgene and additional heterochromatin provided by the extra Y chromosome affects severely the viability of females (female/male ratio 0.26). The effect on viability lends weight to a model whereby dosage compensation is sensitive to heterochromatin.

## Discussion

**Figure 8:**
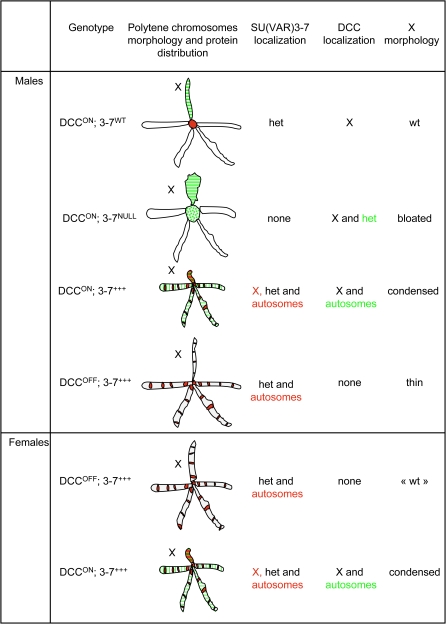
Summary of the interactions between SU(VAR)3-7 and the DCC, and illustration of resulting changes in chromatin conformation and proteins distribution. Tested genotypes are on the left. DCC^ON^ refers to wild type males or to ectopic MSL2-expressing females, DCC^OFF^: homozygous *mle^1^* mutant, 3-7^ +++^: *Su(var)3-7* over-expression context. The drawings compile X chromosome morphological data and immunostainings of MSLs and SU(VAR)3-7. The set of Drosophila salivary gland polytene chromosomes is schematized and the X chromosome indicated. Chromocenters aspect are as described in [33] and [40]. Immunostainings are illustrated as follows: The DCC is represented in green and SU(VAR)3-7 in red. Detailed SU(VAR)3-7 and DCC localization on polytene chromosomes and X chromosome morphology is on the right. “het” stands for heterochromatin. Major changes in protein distribution are highlighted in colour.

Our work reveals a connection between heterochromatin and dosage compensation in Drosophila. We determined that SU(VAR)3-7 is implicated in male X chromosome morphology, in correct distribution of the DCC, in the expression of the dosage compensated *white* gene and in male viability. Figure 8 recapitulates some of the complex interactions between SU(VAR)3-7 and the DCC and illustrates the ability of heterochromatin/DCC balance to affecting chromatin conformation and protein distribution. Our results support a model whereby the activating dosage compensation system in Drosophila is influenced by chromatin silencing factors.

### Male X Chromosome Sensitivity to SU(VAR)3-7

Reduced levels of SU(VAR)3-7 induce bloating of the male X chromosome, whereas increased levels cause condensation of the male X chromosome [33],[40]. Moreover, at high dose, SU(VAR)3-7, normally restricted to heterochromatin, invades preferentially the male X chromosome and, to a lesser extent, the autosomes [40]. These observations led us to investigate the features rendering the male X chromosome particularly sensitive to SU(VAR)3-7. In this paper we have examined the genetic interaction between a gene essential for dosage compensation, *mle*, and *Su(var)3-*7 on the morphology of the male X chromosome. Bloating and shrinking of the X chromosome both require the presence of the DCC, and assembly of the DCC in females is sufficient to make their X chromosomes preferential targets for SU(VAR)3-7, when in excess (Figures 1 and 2). The dosage compensation system is thus responsible for the sensitivity of the male X chromosome to changes in SU(VAR)3-7 amounts. One explanation for the X chromosome sensitivity is that increased levels of H4K16 acetylation induced by the DCC render chromatin of the male X chromosome more accessible to chromatin factors and more sensitive to disturbances than other chromosomes [3],[4],[55]. We cannot exclude the possibility that SU(VAR)3**-**7**-**induced X chromosome defects are indicators of a more general effect of the protein on all chromosomes as described for ISWI: Null mutations in the gene encoding ISWI cause aberrant morphology of the male X chromosome but not of autosomes and females X chromosomes [29], but expression of a very strong dominant negative form of ISWI in vivo leads indeed to decondensation of all chromosomes in both sexes [56]. Nevertheless other data in our work discussed later favour the hypothesis whereby X chromosome defects result from a specific interaction between SU(VAR)3-7 and dosage compensation.

Male X chromosome sensitivity to SU(VAR)3-7 was rather unexpected, as in a wild-type context, in contrast to over-expression conditions, we did not detect preferential binding of SU(VAR)3-7 to the male X chromosome. The absence of detectable SU(VAR)3-7 enrichment on the male X polytene chromosome from third instar larvae may be due either to lack of sensitivity of the immunostaining procedure or to observations made in inappropriate tissues or development stages. Similar puzzling observations have been made for HP1, which is not preferentially seen on the male X polytene chromosomes, although reduced HP1 induces bloating of the male X chromosome [33],[57],[58],[59]. In cultured cells however, a moderate HP1 enrichment was detected with the DamID technique on the male X chromosome and not on the female X chromosomes [60], suggesting that HP1 participates in the structure of the male X chromosome.

### Wild-Type Amounts of SU(VAR)3-7 Are Required for X Chromosome-Restricted Binding of the MSL Proteins

A striking and novel result of this study is that precise wild-type amounts of the heterochromatic protein SU(VAR)3-7 are required to restrict MSLs binding to the X chromosome. In *Su(var)3-7* mutants, we have observed that the MSL proteins are recruited to the chromocenter (Figure 4 and Figure S1). Furthermore, when SU(VAR)3-7 is present in excess, MSLs are massively delocalized from the X chromosome to many sites on autosomes (Figure 5).

We propose two hypotheses. First, the effect of SU(VAR)3-7 on the MSLs distribution is indirect and due to the regulation of the expression of a component of the DCC. Indeed, increased amounts of MSL1 and MSL2 lead to MSLs binding on autosomes and at chromocenter [19],[20],[61], and MSLs delocalization from the X chromosome to autosomes and chromocenter is detectable in *roX1roX* double mutants [22],[23]. A careful regulation of MSLs and *roX* RNAs concentration is therefore important to restrict DCC activity to appropriate targets. In addition, increased levels of MSL2, or of both MSL2 and MSL1, result in a diffuse morphology of the X chromosome [7],[20],[53]. This phenotype resembles the bloated X chromosome of *Su(var)3-7* and *Su(var)2-5* mutants, suggesting that the amounts of MSL2 and MSL1 are downregulated by the heterochromatic proteins. Expression of many euchromatic genes are under the control of the HP1 protein [62],[63],[64],[65], leading us to test whether changes in SU(VAR)3-7 amounts modify the expression of *roXs*, *msl1* and *msl2* or the stability of MSL1 and MSL2. Quantitative RTPCR (Figure 6) and Western blots did not detect significant changes in the amounts of DCC components. In HP1 mutant *msl1* transcription is also not affected [66]. These results speak against the hypothesis of regulation of expression of a DCC component by a SU(VAR)3-7/HP1 complex.

The second hypothesis is that SU(VAR)3-7 modifies the MSLs distribution by changing the chromatin state of the X chromosome and of the pericentric heterochromatin. Changes in chromatin conformation or epigenetic marks could modify affinity of the DCC for entry sites [15],[67]. Demakova et al. [19] and Dahlsveen et al. [15] have described numerous entry sites on the X chromosome, and have suggested a hierarchy of entry sites with different affinities for the DCC. Even cryptic binding sites on autosomes and at the chromocenter are recognized by the DCC in certain conditions. We propose that increasing SU(VAR)3-7 amounts on the X chromosome results in an enrichment of HP1 and H3K9 dimethylation [40], and leads to a more compact heterochromatic-like structure of the X chromosome which then blocks access to the high-affinity entry sites. The free DCC, chased from the X chromosome sites turns toward low-affinity sites present on autosomes, but not toward those embedded into the chromocenter. Indeed, cryptic chromocenter sites become more inaccessible by heterochromatin compaction [40], a phenomenon also responsible for the enhancement of variegation by increased SU(VAR)3-7 levels [40]. Inversely, the absence of SU(VAR)3-7 induces a more relaxed chromatin state at the chromocenter [33], thus increasing affinity of the entry sites embedded into heterochromatin, and allowing MSLs binding at the chromocenter. Similar recruitments of MSLs at heterochromatin have been described in the literature in three situations: in *roX1roX2* mutants [22],[23], in presence of excess of MSL2 [19] and in C-terminal truncated MSL2 mutants [21]. This means that cryptic entry sites present in heterochromatin become more accessible to the MSLs either in a *Su(var)3-7* mutants or if DCC composition is modified. The explanation of heterochromatin affinity for the MSLs remains obscure. On the X chromosome, the *Su(var)3-7* mutation induces the bloated morphology resembling that described as a result of decreased levels of silencing factors as HP1, ISWI and NURF [29],[30],[33], or of increased MSLs levels [20]. Our study and others suggest that male X chromosome morphology depends on the balance between silencing and activating complexes [2],[27],[28],[32]. The simultaneous existence of the repressive SU(VAR)3-7/HP1 proteins and the MSLs complex may provide a set of potential interactions that cumulatively regulate dosage compensation [6].

### Interplay between SU(VAR)3-7 and Dosage Compensation

Several arguments support a role for SU(VAR)3-7 in dosage compensation. Reduced male viability in the progeny of *Su(var)3-7* homozygous females is a first argument for a function played by the protein specifically in males [52]. Our results also show that wild-type amounts of SU(VAR)3-7 are required to cope with increased MSL1 and MSL2 levels. In absence of maternal SU(VAR)3-7 product, the transgenes expressing MSL1 and MSL2 become toxic to males, whereas no lethality is observed with wild-type or half amounts of SU(VAR)3-7 (Table 1). This suggests that SU(VAR)3-7 is required very early in development to counteract an excess of MSL1 or MSL2 activity. Corroborating this effect, we determined that the global amount of heterochromatin affects the viability of females engineered to expressing *msl2*. The presence of the highly heterochromatic Y chromosome kills half of the females expressing *msl2*. As proposed by Weiler and Wakimoto [68],[69], the Y chromosome functions as a sink for heterochromatic factors as SU(VAR)3-7 and HP1 [68],[69]. A Y chromosome added to XX females could sequester heterochromatic proteins, and induce lethality in a context of female dosage compensation. All these data lead to the conclusion that SU(VAR)3-7 is required for the viability of dosage-compensated flies. We propose two explanations: Either SU(VAR)3-7 is required to restrict DCC on the X chromosome, and the lethality induced by the lack of SU(VAR)3-7 is due to the MSLs ectopic activity outside of the X chromosome (at heterochromatin), or SU(VAR)3-7 is required on the dosage compensated X chromosome and, in this case, the *Su(var)3-7* mutant lethality results from malfunctioning of the DCC on the X.

To discriminate between these two hypothesis, we examined expression of X-linked genes in *Su(var)3-7* mutants (Figure S2). Although small changes are visible, the RT-PCR analysis did not allow us to conclude that the lack of SU(VAR)3-7 affects significantly the levels of transcripts of seven X-linked genes. If they exist, changes were indeed expected to be very small. For MSLs mutations, the magnitude of the decrease is very modest considering the severe failure of dosage compensation (around 1.5 [10],[11]). Taking into account that the *Su(var)3-7* mutation induces only 30% lethality among males, expected changes in transcript accumulation are predicted to be even smaller. Moreover, transcripts analysis was done in male larvae and some slight biological variations between our three samples cannot be avoided though great care was taken on samples homogeneity. Finally, normalizing to internal autosomal genes RNA could also introduce a bias [6]. We believe that in our case, quantitative RT-PCR experiment was not the appropriate method to detect very small changes of expression.

In consequence, we have used an alternative system to test the implication of SU(VAR)3-7 on dosage compensation. We have determined the effect of increased or decreased *Su(var)3-7* expression on the dosage compensated expression of the *white* gene carried by P transgenes. Strikingly, we observed that lack and excess of SU(VAR)3-7 decreases the *white* expression specifically in males, and never in females (Figures 3 and 7). This is a strong indication that the wild type dose of SU(VAR)3-7 is required for correct dosage compensated expression of the *white* gene. Interestingly, *Su(var)3-7* over-expression affects *white* expression when the gene is localized on the X chromosome and not on autosomes, although *white* is still partially dosage compensated on autosomes [51]. This may result from the combination of two phenomena: On the X chromosome, excess of SU(VAR)3-7 induces preferential enrichment of heterochromatic silencing proteins and partial loss of MSLs. On autosomes, heterochromatic proteins recruitment is less visible and, in addition, the MSLs are massively present [40] (Figure 5). Consequently the dosage compensation of a P*(white*) transgene linked to the X chromosome is more likely to be perturbed by excess of SU(VAR)3-7 than an autosomal insertion.

In sum, we have revealed in this study a role for SU(VAR)3-7 on global X chromosome morphology with an impact on the distribution of MSLs proteins, thus highlighting the contribution of SU(VAR)3-7 to the intriguing issue of X specific DCC targeting. It appears also that SU(VAR)3-7 is required for the viability of dosage compensated flies and the expression of a dosage compensated X-linked gene, suggesting a puzzling interplay between heterochromatin and the DCC. SU(VAR)3-7 plays a subtle role on dosage compensation: Flies need SU(VAR)3-7, especially the maternal protein, for correct dosage compensation but, at the same time, excess of SU(VAR)3-7 has a negative effect on dosage compensation. Our future interest will focus on the fascinating issue of the molecular nature of heterochromatin/DCC intersection.

## Materials and Methods

### Fly Stocks

*Su(var)3-7* over-expression: heat-shocks were carried out on *Drosophila melanogaster* lines containing the inducible *Su(var)3-7* transgene P(*HA:SuvarFL*) (lines 1A and 4D [48]) and on the *yw^67^* control line. Egg laying was at 25°C for 24 hours, and larvae were incubated at 30°C for two days. Three heat-shocks of 30 minutes at 35°C were then performed each day until adulthood. *Su(var)3-7* mutants: we used the null mutant *Su(var)3-7^R2a8^* [33] raised at 25°C and the hypomorphic *Su(var)3-7^9^* mutant [52] raised at 29°C. P(mini-*white*) stocks were kindly provided by D. Pauli. Flies harbour one P(*UASt* mini-*white*) transgene localized at different locations on the X or on autosomes [70]. The P(*UASt* mini-*white*) transgenes harbor different cDNA sequences that are not expressed due to the lack of GAL4 drivers.

### Fly Crosses

- To test whether reduced SU(VAR)3-7 amounts modify the extent of MSLs spreading around a P[w^+^GMroX1] transgene insertion [49], we crossed ten transgenic y*wroX1^ex6^/Y*;P[w^+^GMroX1] males (from three lines with insertion at 85D, 69C or 79B kindly provided by R. Kelley [49]) to ten wild type (w^1118^) females or *w^1118^; Su(var)3-7^R2a8^* homozygous females. Male larvae were collected for immunostaining and male adults were kept to examine the effect of the lack of maternal SU(VAR)3-7 on *white* expression. Eyes of adult flies from several crosses replica were examined five days after hatching.

- To test the effects of increased SU(VAR)3-7 amounts on *white* expression of P(mini-*white*) transgenes, ten P(mini-*white*) homozygous females were crossed at 25°C either to wild-type males or to males harbouring the transgene that over-expresses *Su(var)3-7* by heat-shock induction [48]. Progeny from both crosses was submitted to daily heat-shocks at 35°C from the third instar larval stage to adulthood or were kept at 25°C for all development. Eyes of adult flies from several crosses replica were examined five days after hatching.

- The genetic crosses shown in Table 1 were realized as follows: Fifteen *w^1118^; Su(var)3-7^R2a8^* homozygous or heterozygous females were crossed at 25°C with fifteen *w/Y; msl2 cn; H83MSL1-H83MSL2/+* males. Progeny *w; msl2 cn/+; Su(var)3-7^R2a8^/+* with (red eyes) or without (white eyes) the H83MSL1-H83MSL2 transgenes was counted. Dramatic difference in males viability was observed depending upon whether or not the mother supplied wild type SU(VAR)3-7 protein in the egg. Similar crosses were done with wild-type and *Su(var)3-9^17^* homozygous females.

- To test the effect of an additional Y chromosome in females that ectopically express MSL2 [7], fifteen wild type (w^1118^) females or *yw; msl1*/*Cyo;* P(*w^+^H83-MSL2*)6I/P(*w^+^H83-MSL2*)6I females were crossed at 25°C either to males bearing a compound X-Y chromosome (*C(1;Y)yw*) or to *w^1118^* males. In the F1, all the females and males were counted without discriminating the presence of the CyO balancer or of the chromosome II with the endogenous *msl1* mutation. In fact 95% the F1 females carry the endogenous *msl1* mutation that supports their survival. Crosses were done several times and the percentage of males and females obtained in each cross is given in Supporting Information Table S1.

### Staining and Immunostaining of Polytene Chromosomes

Orcein stainings of polytene chromosomes were done as in [33]. Procedures for immunostaining were those of Platero et al., [71] with the following modifications: fixation and squashing were done in 4% formaldehyde. Primary antibodies were used at the following dilutions: 1∶10 for anti-SU(VAR)3-7 [35], 1∶400 for anti-HP1 (a gift of Lori Wallrath) and 1∶200 for anti-MSL2 and anti-MSL1 (gifts of M. Kuroda), and for anti-H4K16ac (a gift of Brian Turner). For immunostaining experiments, two independent *Hs-Su(var)3-7* transgene insertions and two independent *Su(var)3-7* mutants have been studied to avoid genetic background effect. For each staining, mutant and wild-type larvae were always treated together; salivary glands were squashed in the same conditions, incubated with the same antibodies preparation, and analyzed in same exposure conditions. Each experiment was done several times.

### Real-Time Quantitative PCR

RNA was isolated from 30 male third instar larvae using Trizol (Invitrogen). After DNase treatment (Ambion DNA free™), one µg of total RNA was used to make cDNA using random hexamers and the Supercript II reverse transcriptase (Invitrogen). Linear real time PCR was performed using Power SYBR Green Master Mix (Applied Biosystems), on a SDS 7900 HT instrument (Applied Biosystems) with the following parameters: 50°C for two minutes, 95°C for ten minutes, and 45 cycles of 95°C 15 secondes−60°C one minute. Each gene was tested with specific primers designed using the program Primer Express v 2.0 (Applied Biosystems) with default parameters. Oligonucleotides sequences will be provided on request. Triplicates of samples and triplicates of PCR were performed and the results obtained for each tested genes were normalized with two or four control genes treated in parallel (tubulin α, RP49, RNA pol II, EFG1). Raw Ct values obtained with SDS 2.2 (Applied Biosystems) were imported in Excel and normalisation factor and fold changes were calculated using the GeNorm method [72]. Real-time PCR and data analysis were performed at the Genomics Platform, NCCR “Frontiers in Genetics” (http://www.frontiers-in-genetics.org/genomics.htm).

### Western Blots

Brains of 20 third instar larvae were dissected in Ringer and resuspended in SDS lysis buffer (1% SDS, 10 mM EDTA, 50 mM Tris-HCl, pH 8) [73]. Proteins in samples were dosed using the BCA™ Protein Assay Kit (Pierce). Samples (5 µg or 10 µg of proteins) were separated on SDS PAGE and transferred on a PVDF membrane (Millipore). Membranes were blocked in TBS, 0.1% tween, 5% dry milk or BSA with α-MSL-1 (1/500) α-tubulin (1/2000) (Sigma T 9026), α-H4K16ac (1/50), α-H3 (1/5000) (Abcam 1791). Membranes were washed with TBS, 0.1% Tween, incubated with secondary antibodies coupled to HRP and revealed by chemiluminescence.

## Supporting Information

Figure S1 DCC components distribution on polytene chromosomes of *Su(var)3-7* mutant. Immunodetection of MSL1 and MSL2 or H4K16ac on wild-type males (WT) or *Su(var)3-7^9/9^* males raised at 29°C (*Su(var)3-7^−/^* ^−^). Arrows indicate the X chromosome and arrowheads show the chromocenter.
(3.00 MB TIF)

Figure S2 Dosage compensation of X-linked genes is not affected in *Su(var)3-7* mutants. RNA levels of seven X-linked genes (*arm*, *BRC*, *CG14804*, *dspt6*, *Gs2*, *mRpL16*, *Pgd*) and two autosomal genes *(tubα* and *RNApolII*) were analyzed by quantitative RT-PCR in homozygous *Su(var)3-7^R2a8^* and wild-type male third instar larvae. Triplicates of samples as triplicates of PCR were realized and the results obtained for each tested genes were normalized with two control genes (Materials and Methods). Bars represent standard deviation from the mean.
(1.00 MB TIF)

Table S1 An extra Y chromosome affects viability of females engineered to expressing *msl2*.
(0.03 MB DOC)
