# Supplementary figures and images for: SU(VAR)3-7 Links Heterochromatin and Dosage Compensation in Drosophila
Source: PLoS Genet. 2008 May 2;4(5):e1000066. doi: 10.1371/journal.pgen.1000066 (PMC2320979; doi:10.1371/journal.pgen.1000066)

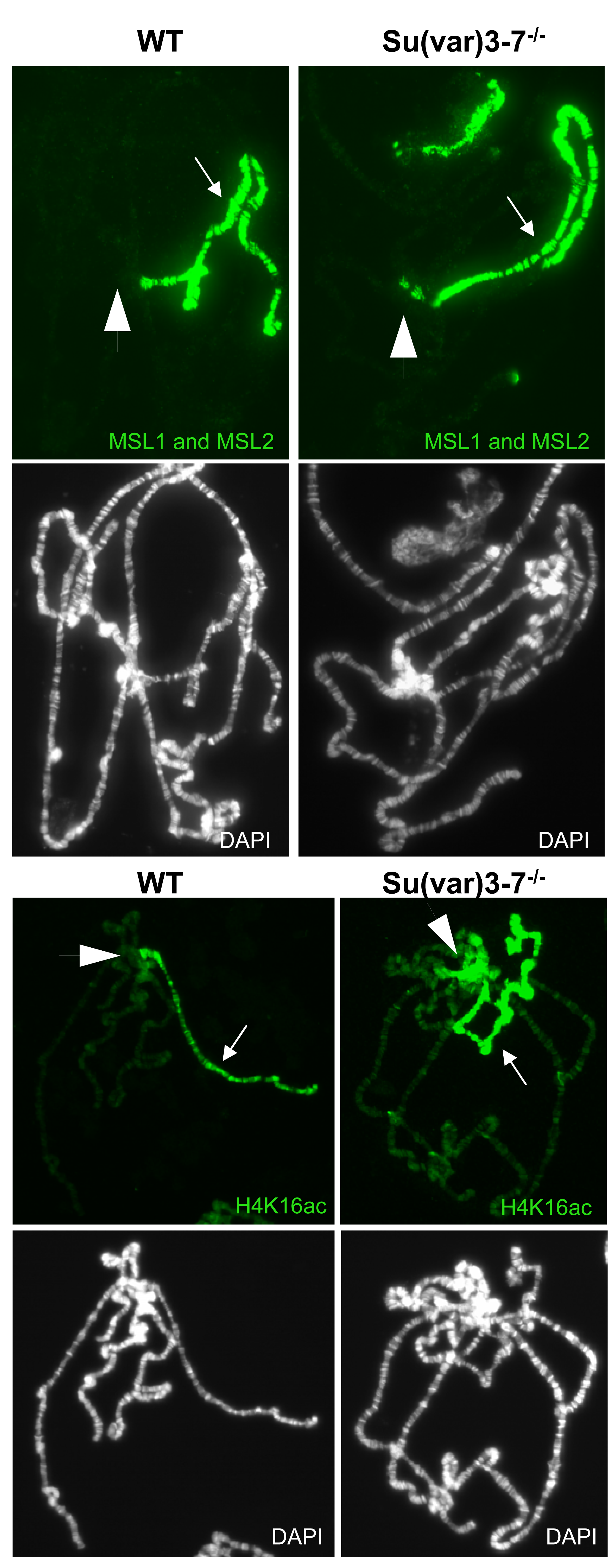

Supplement: Figure S1 — DCC components distribution on polytene chromosomes of Su(var)3-7 mutant. Immunodetection of MSL1 and MSL2 or H4K16ac on wild-type males (WT) or Su(var)3-79/9 males raised at 29°C (Su(var)3-7−/ −). Arrows indicate the X chromosome and arrowheads show the chromocenter. (3.00 MB TIF) [file pgen.1000066.s001.tif]

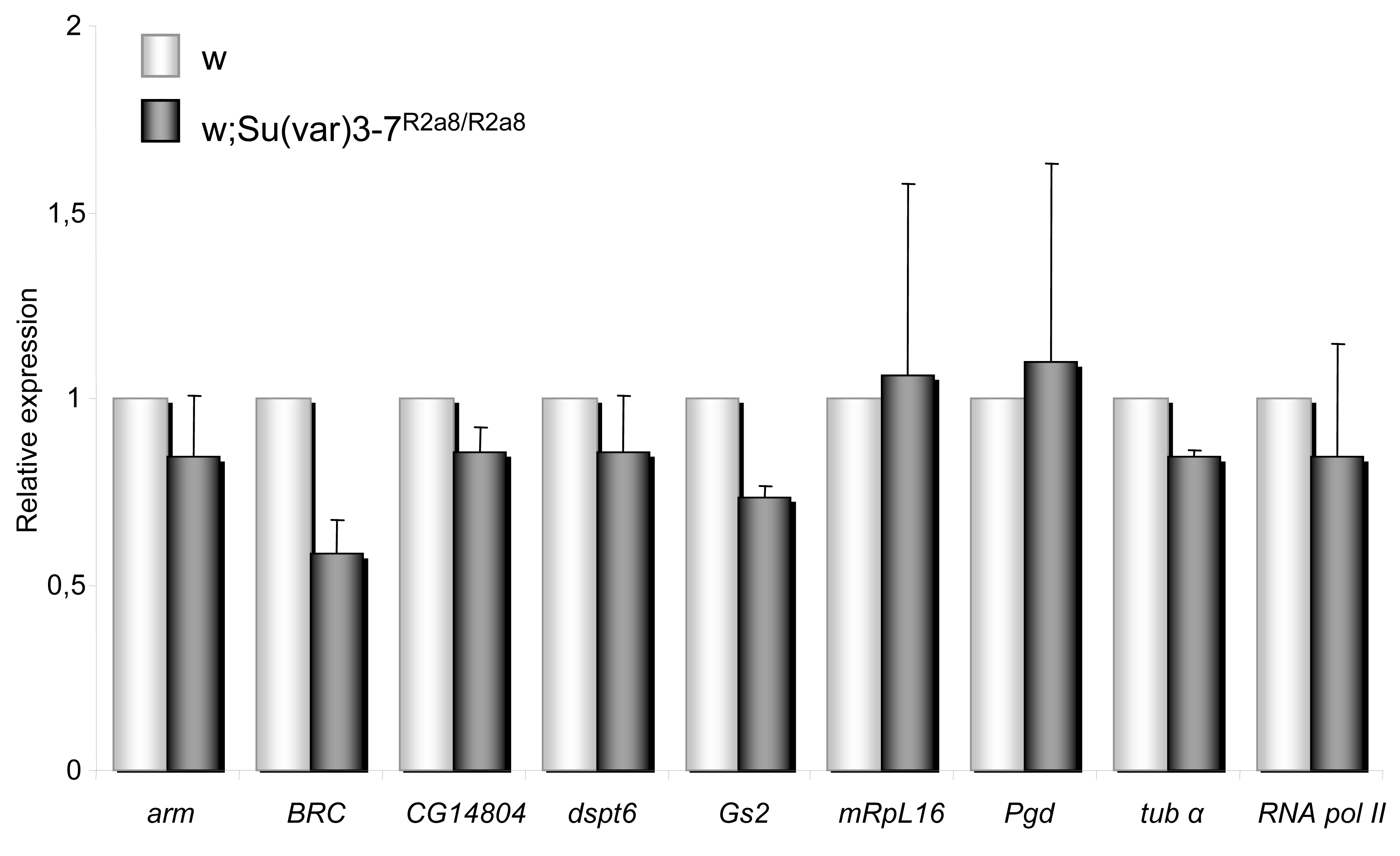

Supplement: Figure S2 — Dosage compensation of X-linked genes is not affected in Su(var)3-7 mutants. RNA levels of seven X-linked genes (arm, BRC, CG14804, dspt6, Gs2, mRpL16, Pgd) and two autosomal genes (tubα and RNApolII) were analyzed by quantitative RT-PCR in homozygous Su(var)3-7R2a8 and wild-type male third instar larvae. Triplicates of samples as triplicates of PCR were realized and the results obtained for each tested genes were normalized with two control genes (Materials and Methods). Bars represent standard deviation from the mean. (1.00 MB TIF) [file pgen.1000066.s002.tif]
