# Supplementary material for: SU(VAR)3-7 Links Heterochromatin and Dosage Compensation in Drosophila
Source: PLoS Genet. 2008 May 2;4(5):e1000066. doi: 10.1371/journal.pgen.1000066 (PMC2320979; doi:10.1371/journal.pgen.1000066)
Supplement: Table S1 — An extra Y chromosome affects viability of females engineered to expressing msl2. (0.03 MB DOC) [file pgen.1000066.s003.doc]

Table S1:

An extra Y chromosome affects viability of females engineered to expressing *msl2*

| **♂** **♀** | **w^1118^** | | **yw ; msl1^26c^/CyO ; H83-MSL2** | |
| --- | --- | --- | --- | --- |
|  | ♀ XX | ♂ XY | ♀ XX | ♂ XY |
| **w^1118^ Y** | 50% (889) | 50% (941) | 49% (540) | 51% (564) |
|  | ♀/♂ = 0.95 | | ♀/♂ = 0.96 | |
|  | ♀ XX^Y | ♂ X0 | ♀ XX^Y | ♂ X0 |
| **C(1;Y)yw** | 42% (644) | 58% (884) | 21% (174) | 79% (670) |
|  | ♀/♂ = 0.72 | | ♀/♂ = **0.26** | |
